# Normal values for urine renalase excretion in children

**DOI:** 10.1007/s00467-014-2855-y

**Published:** 2014-07-25

**Authors:** Agnieszka Rybi–Szumińska, Joanna Michaluk-Skutnik, Barbara Osipiuk-Remża, Anna Kossakowska, Anna Wasilewska

**Affiliations:** Department of Pediatrics and Nephrology, Medical University of Bialystok, Waszyngtona 17, 15-274 Bialystok, Poland

**Keywords:** Age, Blood pressure, Catecholamines, Normative values, Renalase, Urine

## Abstract

**Background:**

The objective of this study was to establish age-dependent values for urinary renalase/creatinine (renalase/Cr) ratio in healthy children and adolescents.

**Methods:**

The study was conducted on a random sample of 157 healthy children and adolescents (0.1–17.9 years) divided into six age groups in 3-year intervals. Urine renalase concentration was measured using an enzyme-linked immunosorbent assay (ELISA) kit (Uscn Life Science, Wuhan, China).

**Results:**

We analyzed median urine renalase/Cr ratio in particular age groups with the use of analysis of variance (ANOVA). Renalase/Cr levels were significantly higher in the youngest children < 3 years in comparison with other age groups (4.07 ng/mg Cr, *p* < 0.05). There was a statistically significant negative correlation between urine renalase/Cr and body mass index (BMI) Z-score (*r* = −0.22, *p* < 0.05) and both systolic (*r* = −0.22, *p* < 0.05) and diastolic (*r* = −0.21, *p* < 0.05) blood pressure. We constructed the reference renalase/Cr percentiles according to age in 3-year intervals.

**Conclusions:**

To the best of our knowledge, this study is the first to present reference values of urine renalase excretion in a healthy pediatric population. Further studies should concentrate on the influence of increased blood pressure or obesity on urine renalase excretion in children and teenagers.

## Introduction

Renalase is a flavin adenine dinucleotide-dependent (FAD-dependent) hormone belonging to the amino-oxidase family that was recently identified by Xu et al. [1]. It is secreted into the blood by the kidney but is also found in heart, skeletal muscles, small intestine, and liver. Renalase can be easily detected in plasma or urine by Western blotting. In the circulation, it resides as a 32-kDa monomer, whereas in urine, in addition to the band of expected size, second, larger doublet (65–75 kDa) has been detected, which can be a result of aggregation or dimerization of the typical monomeric form [1, 2]. Detection of renalase in blood and urine suggests its production by renal tissue, and undetectable levels of the enzyme in the plasma of patients with uremia confirm this hypothesis [1, 2]. Renalase metabolizes circulating catecholamines and thus regulates blood pressure (BP) and sympathetic tone [3, 4]. In vivo observations show that renalase occurs in blood as a precursor and is activated by increased catecholamines levels. Its strongest enzymatic activity is toward dopamine, adrenaline, and noradrenaline [5]. Renalase levels are studied in clinical cardiology and nephrology. Normal values are essential for interpretation of results, especially in pediatric patients. Analysis in our study aimed to establish reference values and upper limits for children and teenagers. Despite many studies in adults, there are still no data concerning serum or urine renalase concentration in the pediatric population. The aim of this study was to establish age-dependent values for urinary renalase concentration in healthy children and adolescents.

## Participants and methods

The study was conducted on a random sample of 157 healthy children and adolescents (78 boys; 79 girls) aged 8.5 (0.1–17.9) years. Materials and data were obtained from participants in the (OLAF) study and from healthy children of hospital staff members. Informed consent was obtained from parents of all participants and from children > 16 years of age. The study protocol was approved by Local Committee of Bioethics, Medical University of Bialystok, while the OLAF study was approved by the Children’s Memorial Health Institute Ethics Committee. Past and present medical history was taken from parents. Body weight and height were measured using a balance-beam scale and pediatric wall-mounted stadiometer; body mass index (BMI) was calculated as weight (in kilograms) divided by the square of height (meters squared). Age-specific reference values for BMI were generated by the lambda, mu, sigma method (LMS) method [6]. LMS values were taken from the OLAF study published by Kulaga et al. [7]. Blood pressure was measured using a validated oscillometric device (Datascope Accutor Plus) with the participant in a sitting position. Three measurements were taken in 3-min intervals, and the arithmetical average was used for statistical analysis. Anthropometric methods have been described thoroughly in OLAF study publications [8, 9]. BMI values are expressed as Z-scores. Inclusion criteria were healthy children and adolescents aged 0.1–18 years. Exclusion criteria were any signs of infection, chronic diseases, and medications that influence renal function or interfere with BP values.

After an overnight fast, first morning-voided urine samples were collected, transported to the laboratory within 4 h, and frozen at −80 °C. Urine renalase concentration was measured using the enzyme-linked immunosorbent assay (ELISA) kit (Uscn Life Science, Wuhan, China) according to manufacturer’ instructions and expressed as renalase/creatinine ratio (renalase/Cr) in ng/mg creatinine. Mean intra- and interassay coefficients of variation for renalase were CV <10 % and CV <12 %, respectively. The detection limit was 3.12–200 ng/ml.

Data were analyzed with Statistica program (version 10.0, StatSoft, Tulsa, OK, USA), and the Kolmogorov–Smirnov test was used to determine normality of variables. Discrete variables were expressed as counts (percentage) and continuous variables as median and quartiles, unless stated otherwise. The comparison between the two groups was done using chi-square and Fisher exact tests for categorical variables and *t* test for continuous variables for normally distributed data, or Mann–Whitney or analysis of variance ;(ANOVA) tests for nonnormally distributed data. Correlations between urine renalase/Cr and other variables were evaluated by Pearson’s or Spearman’s test, as appropriate, in both groups. *P* < 0.05 was considered statistically significant. Age-specific reference values for renalase/Cr ratio were generated by LMS [10] using LMSChartMaker software, which characterizes the distribution of a variable by its median, CV, and skewness (L) required to transform data to normality [11]. Centile curves for age were obtained as:

$$ {C}_{100\alpha}\left(\mathrm{t}\right)= M(t){\left[1+ L(t)\; S(t) Z\alpha \right]}^{1/ L(t)} $$

where *Zα* is the normal equivalent deviate for tail area α, and *C*_100α_ (t) is the centile corresponding to *Zα*.

## Results

The study group of 157 individuals was divided into six age groups in 3-year intervals: 0.1–2.9, 3–5.9, 6–8.9, 9–11.9, 12–14.9, and 15–18 years old; 50.3 % were and 49.7 % boys of similar age (*p* > 0.05). There were no statistically significant differences in BMI, BMI Z-scores, and systolic (sBP) or diastolic (dBP) BP measurements between girls and boys (*p* > 0.05). Median values of renalase/Cr were similar in both groups (*p* > 0.05). Group characteristics are shown in Table 1.

**Table 1:** Characteristic of study participants

Variable	Female *N* = 79	Male *N* = 78	*P* value
	Median (Q1, Q3)	Median (Q1,Q3)	
Age (years)	8 (5.5–10.9)	8.85 (5–12.2)	NS
BMI	17.31 (14.99–21.3)	18.46 (16.37–21.08)	NS
BMI Z-score	0.1 (−1.02–1.52)	0.62 (0.18–1.51)	NS
Urine renalase/Cr (ng/mg Cr)	160 (89–385)	246 (110–430)	NS
sBP (mmHg)	108 (103–115)	108 (102–119)	NS
dBP (mmHg)	63 (58–70)	65 (60–68)	NS

*BMI* body mass index, *Cr* creatinine, *sBP* systolic blood pressure, *dBP* diastolic blood pressure, *Q* quartile, *NS* not significant

**Fig. 1:**
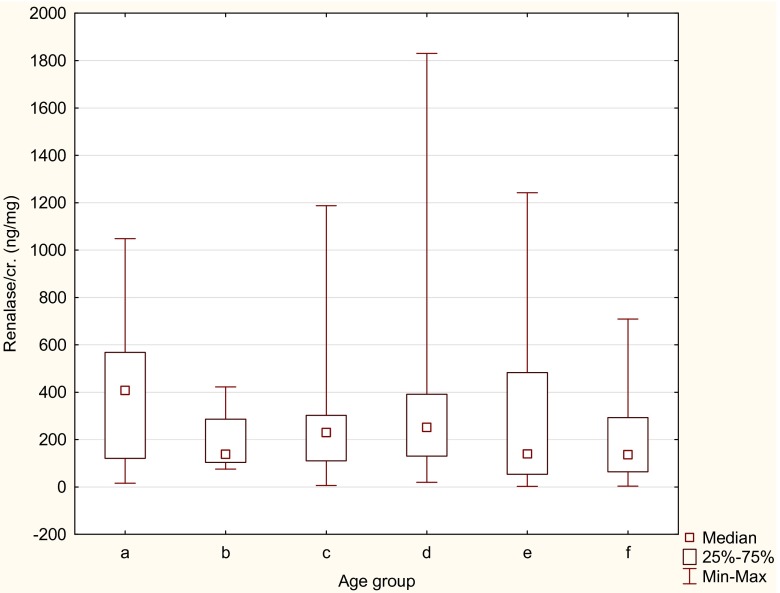
Comparison of urine renalase/creatinine ratio between six age groups (analysis of variance chi square = 11.19, *p* < 0.05). Age groups: **a** 0.1–2.9 years, **b** 3–5.9 years, **c** 6–8.9 years, **d** 9–11.9 years, **e** 12–14.9 years, **f** 15–17.9 years

We observed statistically significant negative correlation between urine renalase/Cr and age (*r* = −0.16, *p* < 0.05). Therefore, we assessed urine renalase/Cr in particular age groups in 3-year intervals. The highest values were found in the youngest children (0.1–2.9 years), and we observed a decrease in renalase urine concentration with age. Statistically significant difference between the youngest group and all other groups was found. Additionally, significant increase in urine renalase/Cr was found in children aged 9–11.9 years. Figure 1 presents statistically significant differences in median concentration of urine renalase/Cr in particular age groups (ANOVA, *p* < 0.05) The highest levels were in children <3 years (407 ng/mg Cr) and the lowest in the oldest children >15 years (136 ng/mg Cr).

Reference percentiles for renalase/Cr without stratification for gender were constructed in 3-year intervals without skewness. The Box–Cox power transformations *L* for height were set to 1 at all age groups. Renalase/Cr edf parameters were L3M5S3. Results are given in Table 2.

**Table 2:** Urine renalase/creatinine ratio reference values by age intervals (ng/mg)

Edf:				Centiles (urine renalase/ cr.)						
	3	5	3	3 c	10 c	25 c	50 c	75 c	90 c	97 c
Age (years)	L	M	S	−2.0001	−1.3334	−0.6667	0	0.6667	1.3334	2.0001
0–2.9	38.08	245.26	81.3	19.42	60.58	133.65	245.26	401.46	607.82	869.55
3–5.9	30.97	190.17	85.18	16.88	46.91	101.84	190.17	320.88	503.27	746.99
6–8.9	27.81	211.09	93.38	15.14	45.78	106.56	211.1	374.85	614.94	950.09
9–11.9	26.01	211.71	105.08	10.11	37.06	97.69	211.71	402.95	699.21	1132.15
12–14.9	24.12	156.33	113.71	5.79	23.68	67.68	156.33	313.62	569.13	958.2
15–17.9	21.60	99.51	120.89	3.25	13.72	41.05	99.52	209.21	396.78	696.15

**Fig. 2:**
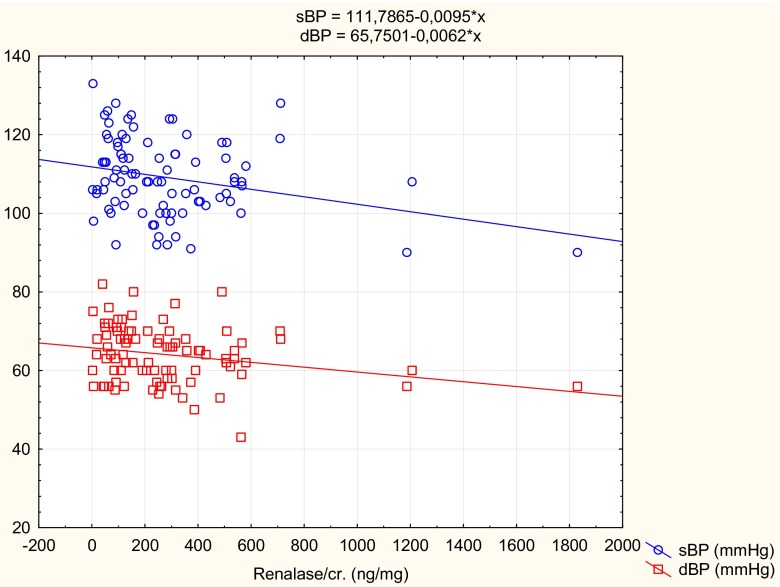
Correlation between urine renalase/creatinine ratio and systolic blood pressure (*sBP*) (*blue line*) and between urine renalase/creatinine ratio and diastolic blood pressure (*dBP*) (*red line*)

We also assessed the potential relationship between urine renalase/Cr and parameters of physical development and BP values. There was a significantly negative correlation between urine renalase/Cr and BMI Z-score (*r* = −0.22, *p* < 0.05) and both systolic and diastolic BP (*r* = −0.22, *p* < 0.05 and *r* = −0.21, *p* < 0.05, respectively) (Fig. 2).

## Discussion

This study provides normative ranges of urine renalase/Cr in designated age groups in a pediatric population. Overall, mean participant age was 8.52 ± 4.76 years, and the sample was nearly equally divided between boys and girls. Differences in patterns of urine renalase levels were not found based on gender. There was a significant negative correlation between renalase/Cr and age, with levels being higher in the youngest children. In reviewing the literature, no data was found on the association between urine renalase excretion and age in the pediatric population; however, there are studies concerning catecholamine evaluation in healthy children and adolescents. Nunez et al. revealed significant age-related differences in urine concentration of dopamine, adrenaline, and noradrenaline [12]. The highest catecholamine levels were observed in children < 2 years old. Li et al. [5] reported that renalase could be activated and secreted into the blood by infusion of catecholamines and/or a transient increase in BP. When renalase was activated, it degraded plasma catecholamines and thus caused a significant drop in BP [5]. Since renalase circulates as a proenzyme and an increase in catecholamine concentrations is an activating trigger, it seems obvious that higher concentrations of dopamine, adrenaline, and noradrenaline may explain elevated renalase levels. Surprisingly, we also found increases in urine renalase/Cr in children aged 9–11.9 years. It seems possible that these results are due to puberty; however, we cannot exclude the possibility that our relatively low sample size might have some influence on this observation.

The source of urine renalase is not fully documented. Renalase is secreted into the bloodstream, and its levels are regulated by three key factors: renal function, renal perfusion, and catecholamine levels. Kidney tissue is the main source of renalase, which is the only enzyme with a potent hydrolytic activity toward catecholamines [5]. In renal tissue, renalase is primarily detected in renal tubular epithelial cells, mesangial cells, and podocytes [13]. Wang et al. showed that only tubular epithelial cells secreted renalase into supernatant, suggesting that these cells are the primary renalase-secreting cells in the kidney [14]. Previous studies indicated that decreased renalase concentration was associated with increased levels of circulating catecholamines and elevated BP [15]. In this study, we also showed a significantly negative correlation between urine renalase/Cr and both systolic and diastolic BP. However, we correlated BP values, not percentiles, so we cannot exclude the influence of age on our results. Nevertheless, our findings are in agreement with Wu et al. [16], who revealed that in the renalase knockout mouse model, lack of endogenous renalase increased sBP and dBP [16]. It was also shown that blood renalase levels were inversely correlated with sBP in patients with resistant hypertension [17, 18].

On the other hand, renalase was positively correlated with dBP in patients wtih type 2 diabetes, though these observations were focused on serum renalase concentration [19] in patients with diabetic nephropathy, which obviously caused elevated BP. Controversially, both renalase and BP levels were higher in patients with a severe kidney insufficiency (serum Cr > 1.5 mg/dl).

The more surprising correlation in our study was with BMI Z-score. No data were found in the literature regarding this relationship; however, a possible explanation may be the coexistence of higher BP values with higher BMI Z-score. Future study investigating the role of renalase in obesity would be very interesting.

## Conclusion

To the best of our knowledge, this is the first study to present reference values of urine renalase excretion in a pediatric population. Although the study is based on a small number of participants, it is worth noting that reference values were determined in a group of healthy children. Further studies should concentrate on the influence of increased BP or obesity on urine renalase excretion in children and teenagers. The most important limitation in this study is that we measured urine renalase concentration using a commercially available assay, and as renalase is an enzyme, its activity should be measured, and assessing its expression only does not ascertain definitively if the protein is active.
